# Cognitive and Behavioral Skills Exercises Completed by Patients with Major Depression During Smartphone Cognitive Behavioral Therapy: Secondary Analysis of a Randomized Controlled Trial

**DOI:** 10.2196/mental.9092

**Published:** 2018-01-11

**Authors:** Toshi A Furukawa, Masaru Horikoshi, Hirokazu Fujita, Naohisa Tsujino, Ran Jinnin, Yuki Kako, Sei Ogawa, Hirotoshi Sato, Nobuki Kitagawa, Yoshihiro Shinagawa, Yoshio Ikeda, Hissei Imai, Aran Tajika, Yusuke Ogawa, Tatsuo Akechi, Mitsuhiko Yamada, Shinji Shimodera, Norio Watanabe, Masatoshi Inagaki, Akio Hasegawa

**Affiliations:** ^1^ Department of Psychiatry and Cognitive-Behavioral Medicine Nagoya City University Graduate School of Medical Sciences Nagoya Japan; ^2^ Center of Cognitive-Behavior Therapy National Center of Neurology and Psychiatry Kodaira Japan; ^3^ Center to Promote Creativity in Medical Education Kochi Medical School Kochi University Nankoku Japan; ^4^ Department of Neuropsychiatry Toho University School of Medicine Tokyo Japan; ^5^ Department of Psychiatry and Neurosciences Hiroshima University Graduate School of Biomedical & Health Sciences Hiroshima Japan; ^6^ Department of Psychiatry Hokkaido University Graduate School of Medicine Sapporo Japan; ^7^ Harimayabashi Clinic Kochi Japan; ^8^ Hokudai-dori Mental Health Clinic Sapporo Japan; ^9^ Shiki Clinic Nagoya Japan; ^10^ Narumi Himwari Clinic Nagoya Japan; ^11^ Department of Health Promotion and Human Behavior Kyoto University Graduate School of Medicine/School of Public Health Kyoto Japan; ^12^ National Institute of Mental Health National Center of Neurology and Psychiatry Kodaira Japan; ^13^ Department of Psychiatry Kochi Medical School Kochi University Nankoku Japan; ^14^ Okayama University Hospital Okayama Japan; ^15^ Advanced Telecommunications Research Institute International Kyoto Japan

**Keywords:** major depressive disorder, smartphone, cognitive therapy, telemedicine

## Abstract

**Background:**

A strong and growing body of evidence has demonstrated the effectiveness of cognitive behavioral therapy (CBT), either face-to-face, in person, or as self-help via the Internet, for depression. However, CBT is a complex intervention consisting of several putatively effective components, and how each component may or may not contribute to the overall effectiveness of CBT is poorly understood.

**Objective:**

The aim of this study was to investigate how the users of smartphone CBT use and benefit from various components of the program.

**Methods:**

This is a secondary analysis from a 9-week, single-blind, randomized controlled trial that has demonstrated the effectiveness of adjunctive use of smartphone CBT (Kokoro-App) over antidepressant pharmacotherapy alone among patients with drug-resistant major depressive disorder (total n=164, standardized mean difference in depression severity at week 9=0.40, J Med Internet Res). Kokoro-App consists of three cognitive behavioral skills of self-monitoring, behavioral activation, and cognitive restructuring, with corresponding worksheets to fill in. All activities of the participants learning each session of the program and completing each worksheet were uploaded onto Kokoro-Web, which each patient could use for self-check. We examined what use characteristics differentiated the more successful users of the CBT app from the less successful ones, split at the median of change in depression severity.

**Results:**

A total of 81 patients with major depression were allocated to the smartphone CBT. On average, they completed 7.0 (standard deviation [SD] 1.4) out of 8 sessions of the program; it took them 10.8 (SD 4.2) days to complete one session, during which they spent 62 min (SD 96) on the app. There were no statistically significant differences in the number of sessions completed, time spent for the program, or the number of completed self-monitoring worksheets between the beneficiaries and the nonbeneficiaries. However, the former completed more behavioral activation tasks, engaged in different types of activities, and also filled in more cognitive restructuring worksheets than the latter. Activities such as “test-drive a new car,” “go to a coffee shop after lunch,” or “call up an old friend” were found to be particularly rewarding. All cognitive restructuring strategies were found to significantly decrease the distress level, with “What would be your advice to a friend who has a similar problem?” found more helpful than some other strategies.

**Conclusions:**

The CBT program offered via smartphone and connected to the remote server is not only effective in alleviating depression but also opens a new avenue in gathering information of what and how each participant may utilize the program. The activities and strategies found useful in this analysis will provide valuable information in brush-ups of the program itself and of mobile health (mHealth) in general.

**Trial Registration:**

Japanese Clinical Trials Registry UMIN CTR 000013693; https://upload.umin.ac.jp/cgi-open-bin/ctr_e/ ctr_view.cgi?recptno=R000015984 (Archived by WebCite at http://www.webcitation.org/6u6pxVwik)

## Introduction

Cognitive behavioral therapy (CBT) is the psychotherapy with the strongest evidence base for the treatment of depression [1-3]. CBT is indeed the only psychotherapy that has been shown to beat the pill placebo condition, the gold standard control condition in the evaluation of medical interventions [4]. It has also been demonstrated to show comparable efficacy as antidepressant pharmacotherapy, which is the mainstay of the treatment for major depression today [5].

The broad umbrella term of CBT, however, now subsumes various and different behavioral and cognitive skills such as self-monitoring, behavioral activation, cognitive structuring, assertion training, structured problem solving, mindfulness, and others [6]. The relative contributions of these various components to the overall efficacy of CBT remain uncertain and debated [7-9]. So-called dismantling studies or component studies to disentangle individual constituents of broadly conceived CBT have been largely underpowered and inconclusive, as each study can only examine the value of adding one particular component in question in a relatively limited number of patients [10,11]. Another major issue of such studies is whether the intended components are actually administered by the therapists and received by the patients, although more recent trials attempt to assure their delivery through audio or video recordings.

The development of information and communication technologies, however, is opening new opportunities to monitor the delivery of CBT skills and to study differential contribution of various components of CBT. The CBT itself can be delivered remotely [12], and the patients’ progress can be remotely monitored [13,14]. Ecological momentary assessment or experience sampling enables more fine-tuned follow-up of patients’ usage of and responses to the program [15,16]. We have developed a smartphone CBT app, named Kokoro-App (*kokoro* means *mind* in Japanese), with the integrated Kokoro-Web secure server to which all the activities of the patients with the app are uploaded. Kokoro-App teaches three distinctive CBT skills, namely self-monitoring, behavioral activation, and cognitive restructuring and provides interactive worksheets that the patients can fill in for each task.

The effectiveness of the system was demonstrated in a randomized controlled trial (RCT) comparing antidepressant medication switch plus Kokoro-App against antidepressant medication switch alone among patients who had been unresponsive to one or more antidepressants: the effect size of the intervention was a standardized mean difference of 0.40 in depression severity as measured by masked assessors (*P*<.001) [17]. This study aims to examine how the patients used the smartphone CBT app during the trial and to investigate what use characteristics differentiated the more successful users of the CBT app from the less successful ones.

## Methods

### Study Design

The original study was a 9-week, multicenter, parallel-group RCT comparing antidepressant medication switch plus smartphone CBT against medication switch alone among patients with antidepressant-resistant depression [17] (Japanese Clinical Trials Registry UMIN CTR 000013693). A total of 164 patients who had not responded to one or more antidepressants at adequate dosage for 4 or more weeks [18] were randomized 1:1 to the intervention or the control arm. The RCT has been registered in the Japanese trials registry (UMIN CTR 000013693).

The randomized comparison showed that the adjunctive use of smartphone CBT brought about 2.5 (95% CI 1.2-3.7, *P*<.001) points greater reduction in the Patient Health Questionnaire-9 (PHQ-9) [19] scores and 4.1 (95% CI 1.5-6.6, *P*=.002) points greater reduction in the Beck Depression Inventory-II [20] scores after 9 weeks [17]. This study focuses on the 81 patients who were randomized to the smartphone CBT arm and describes and analyzes the patients’ use of Kokoro-App.

### Kokoro-App

**Figure 1:**
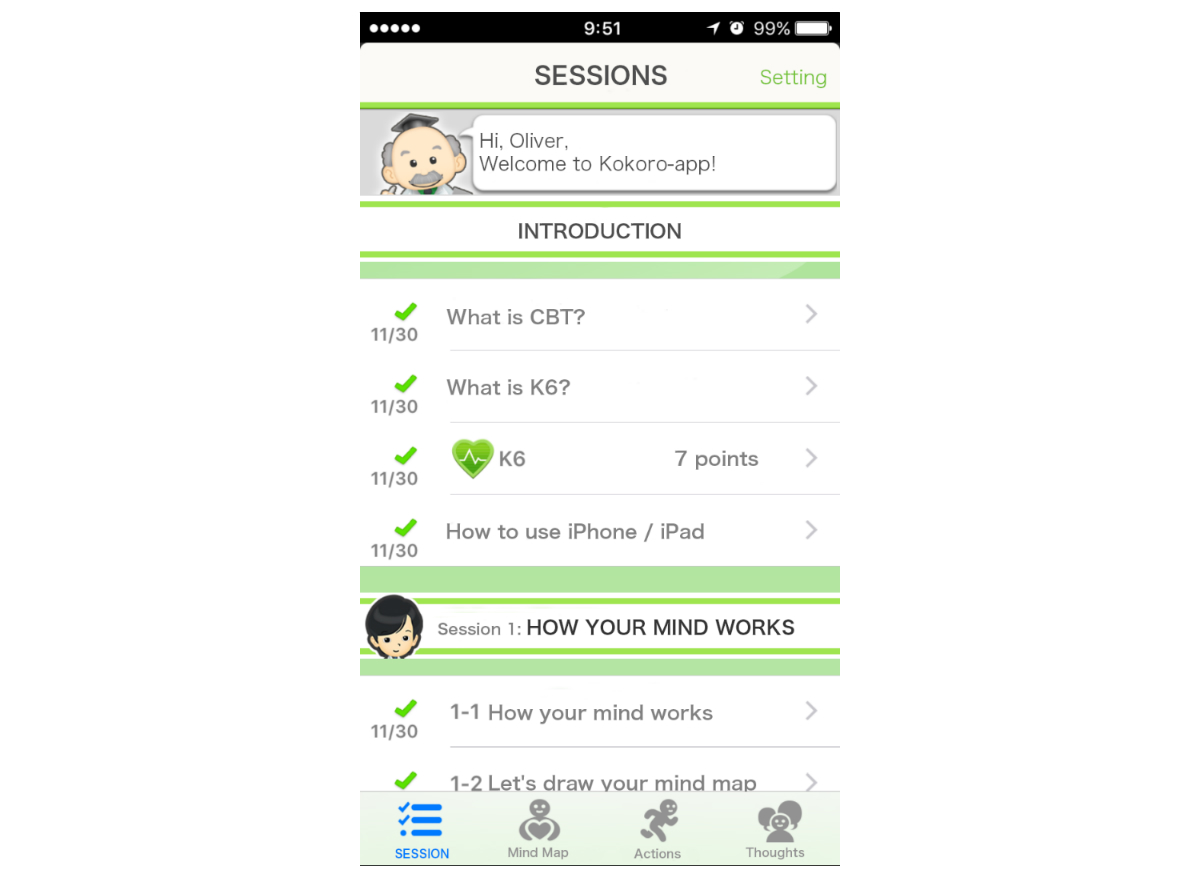
A screenshot from Kokoro-App.

Kokoro-App is a smartphone CBT app and consists of four parts: sessions, mind maps, actions, and thoughts (Figure 1).

There are eight sessions in which several cartoon characters provide psychoeducation through easy but fun conversations. First, the welcome session explains CBT, as well as how to use iPhone and Siri (voice recognition on iPhone). Sessions 1 and 2 explain how to self-monitor one’s reactions to various situations according to the cognitive behavioral model. The sessions introduce mind maps in which the patient can enter details of the situation and his reactions to it in terms of emotion and its degree, automatic thoughts, bodily reactions, and behaviors. The patient chooses between four emotions of sad or depressed, anxious or worried, angry, and happy and rates its strength in five grades between 0 and 5.

Sessions 3 and 4 explain behavioral activation according to two principles of “When the body moves, so does the mind” and “Start small and near.” When the patient clicks on actions, lists of candidate activities for behavioral activation personal experiments pop up. The candidates are categorized by the usual time they require to complete into (1) less than 5 seconds, (2) less than 5 min, (3) less than 60 min, and (4) 60 min or more. The patient chooses a candidate and rates his expected mastery and pleasure levels. When the patient completes the personal experiment, he can enter his achieved mastery and pleasure levels. The patient can also enter his own personal experiment task. After his own experiments, the patient can recommend certain activities by clicking on “Nice!” button and the number of “Nice!”s will be shared by all the patients.

Sessions 5 and 6 explain cognitive restructuring. After providing a rationale for cognitive restructuring, the app provides four interactive items to guide the patient to alternative thoughts. The patient first picks up a mind map to work on. The first item, “fact glasses,” asks classic questions about evidence for and evidence against the automatic thought, such as “What facts do you have to support this thought?” and “What facts are there that do not support this thought?” Then the item combines the patient's answers automatically and says, “So you believe XXX but YYY. If you think this way, how do you feel now?” and asks the patient to rerate his feeling. 

**Figure 2:**
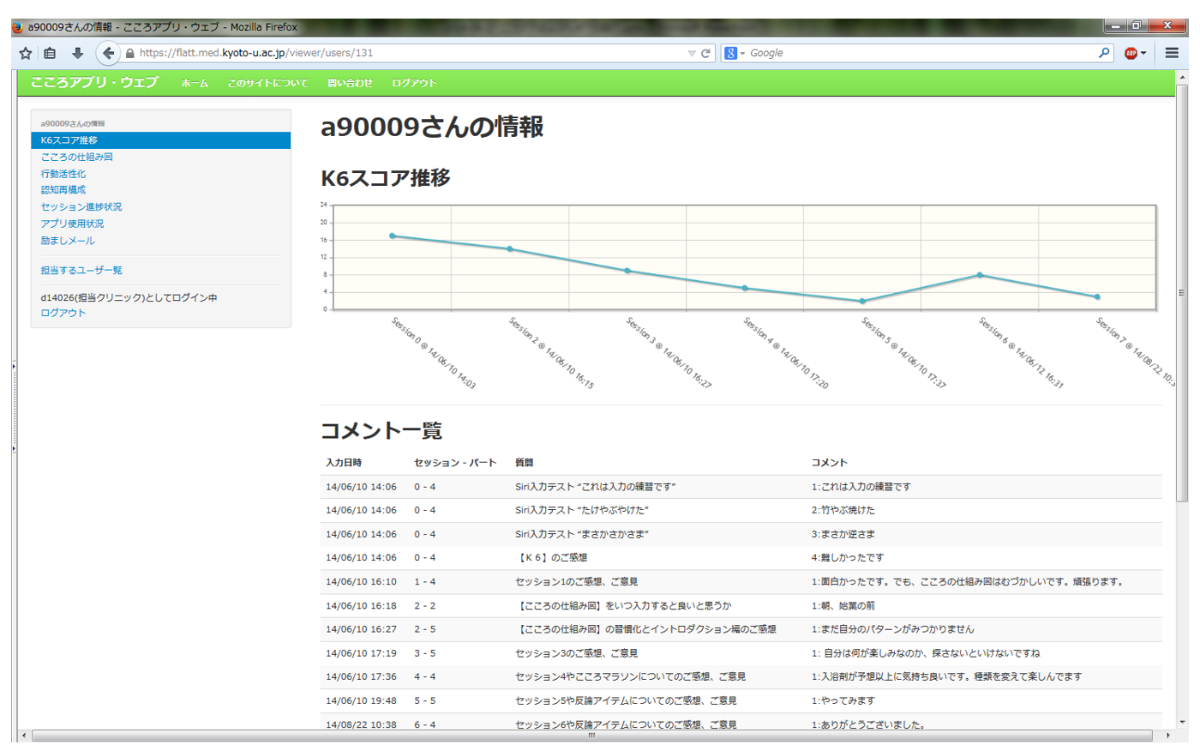
A screenshot from Kokoro-Web.

The second item is called “% Calculator,” which does similar things as the fact glasses. It asks, “How confident are you in your thought AAA?” then lets the patient choose between 1% and 99%. Then it asks, “So you think your thought AAA is 99% correct. But then what can there be in your other 1%?” The patient will then answer XXX, and the item will then ask, “So if you think XXX, how would you feel now?” The third item is “friend’s call.” The item says, “Ring, ring, ring. You have just received a phone call from your best friend, saying AAA. What advice would you give to her?” The rest is the same. The last item is “What-now microphone.” The item goes, “Let’s just suppose that your thought AAA is true. If so, what can be done now?” The patient will then make an action statement, and the item will then ask, “What if you did do XXX, how would you be feeling?”

The epilogue session summarizes all the previous sessions and also provides tips for relapse prevention.

Each session is expected to take 1 week. The new session can be opened only a week after the last session was started and after one homework has been completed.

### Kokoro-Web

All the activities of the patient with Kokoro-App are uploaded to the central server and can be viewed on Kokoro-Web (Figure 2) by the patient, as well as by his treating physician. Kokoro-Web was developed seamlessly and integratively with Kokoro-App. The communication between the app and the server through the Internet was certificated by Secure Sockets Layer.

### Statistical Analyses

We first present the descriptive details of how the patients utilized Kokoro-App. The continuous outcomes are summarized by mean and SD and the dichotomous outcomes by number and percentage.

We next subdivide the patients into beneficiaries and nonbeneficiaries from Kokoro-App at the median change score of the PHQ-9 and compare each group’s use of the Kokoro-App. Because the same patient contributed a variable number of mind maps, behavioral activations, or cognitive restructurings to account for the within-person clustering effect, we used the mixed effects model where appropriate. Given the observational and hypothesis-generating nature of this study, we set the threshold for statistical significance for each comparison at nominal *P*<.05 throughout. We used STATA (StataCorp) version 14.

## Results

### Patient Characteristics

Table 1 summarizes the baseline demographic and clinical characteristics of the cohort. Patients were typically around 40 years of age, had some higher education, slightly more than half were in some employment, and slightly less than half were married. They had had several depressive episodes, had been in the current depressive episode for almost 2 years, and were in moderately to severely depression at baseline.

**Table 1:** Baseline demographic and clinical characteristics of the cohort.

Characteristics			Mean SD^a^ or n (%)
**Demographic**			
	Age (years), mean (SD)		40.2 (8.8)
	Women, n (%)		46 (57)
	Education (years), mean (SD)		14.6 (2.5)
	**Employment status**		
		Employed full-time, n (%)	34 (42)
		Employed part-time, n (%)	7 (9)
		On medical leave, n (%)	21 (26)
		Housewife, n (%)	6 (7)
		Student, n (%)	0 (0)
		Retired, n (%)	0 (0)
		Not employed, n (%)	13 (16)
	**Marital status**		
		Single, never married, n (%)	34 (42)
		Single, divorced, separated or widowed, n (%)	13 (16)
		Married, n (%)	34 (42)
**Clinical**			
	Age of onset at first episode (years), mean (SD)		31.8 (10.8)
	Number of previous depressive episodes, mean (SD)		3.4 (4.9)
	Length of current episode (months), mean (SD)		24.2 (46.3)
	PHQ-9^b^ at baseline, mean (SD)		13.5 (5.5)
	BDI-II^c^ at baseline, mean (SD)		28.2 (11.2)

^a^SD: standard deviation.

^b^PHQ-9: Patient Health Questionnaire-9.

^c^BDI-II: Beck Depression Inventory 2nd edition.

### Kokoro-App Use Statistics

Table 2 shows the use statistics of Kokoro-App by the 81 patients.

On average, the patients completed 7.0 out of 8 sessions; it took them 10.8 days to complete one session, during which they spent 62 min on the app reading the sessions and also completing their respective homework (self-monitoring, behavioral activation, or cognitive restructuring).

They filled in 10 mind maps, more often for sad or depressed, or anxious or worried feelings but also for angry or happy feelings. They performed 14 behavioral activation personal experiments through which they had anticipated and achieved moderate levels of mastery and pleasure. With respect to cognitive restructuring, they generated an average of six alternative thoughts using fact glasses, % Calculator, friend’s call, or what-now microphone almost equally frequently.

### Behavioral Activations

We analyzed the behavioral activations completed by the patients according to their frequencies, the level of mastery and pleasure they achieved, and how unexpectedly good they were.

The most frequently chosen behavioral activations were, in the descending order, “Listen to favorite music,” “Read books and magazines,” “Brew and drink coffee,” and so on (Table 3). The levels of mastery or pleasure expected or achieved were typically in the range 3 to 5 on a scale of 0 to 10.

However, when selected for the levels of achieved mastery or pleasure, very different sets of activities emerged (Tables 4 and 5). These tables are limited to such activities that were reported at least three times. Activities that achieved very high levels of mastery or pleasure included “Test-drive a new car,” “Go to a coffee shop after lunch,” “Call up an old friend,” and “Exercise.”

**Table 2:** Use statistics of Kokoro-App.

Use statistics			Mean (SD^a^), range, and median
**Overall**			
	Sessions completed, mean (SD), range		7.0 (1.4), 1-8
	Days taken to complete one session, mean (SD), range		10.8 (4.2), 6.3-31
	Actual time (min) per session, mean (SD), range, median		62.3 (96.30), 0-677, 39
**Self-monitoring**			
	Mind maps, no. completed per person, mean (SD), range		10.4 (10.5), 0-45
	**Mind maps, no. completed per person, by emotion**		
		Sad or depressed, mean (SD), range	3.2 (3.7), 0-16
		Anxious or worried, mean (SD), range	3.0 (3.3), 0-18
		Angry, mean (SD), range	2.4 (3.4), 0-16
		Happy, mean (SD), range	1.9 (3.1), 0-20
	**Level of emotion recorded (on a scale of 0-5)**		
		Sad or depressed, mean (SD)	3.4 (1.3)
		Anxious or worried, mean (SD)	3.5 (1.3)
		Angry, mean (SD)	3.5 (1.3)
		Happy, mean (SD)	3.2 (1.3)
**Behavioral activation**			
	Behavioral activations, no. completed per person, mean (SD), range		13.8 (17.3), 0-118
	**Level of mastery or pleasure by behavioral activation (on a scale of 0-10)**		
		Mastery expected, mean (SD)	4.5 (2.8)
		Mastery achieved, mean (SD)	4.3 (3.0)
		Pleasure expected, mean (SD)	4.8 (2.7)
		Pleasure achieved, mean (SD)	4.7 (2.9)
**Cognitive restructuring**			
	Cognitive restructuring, no. completed per person, mean (SD), range		6.2 (6.3), 0-31
	**Cognitive restructuring items used, per person**		
		Fact glasses, mean (SD), range	2.0 (2.0), 0-11
		% Calculator, mean (SD), range	1.5 (1.8), 0-10
		Friend’s call, mean (SD), range	1.5 (1.6), 0-7
		What-now microphone, mean (SD), range	1.4 (1.5), 0-7

^a^SD: standard deviation.

**Table 3:** Behavioral activations: top 10 activities in terms of frequency and their mastery or pleasure levels.

Activity	Frequency (number of reports)	Mastery		Pleasure	
		Expected Mean (SD^a^)	Achieved Mean (SD)	Expected Mean (SD)	Achieved Mean (SD)
Listen to favorite music	95	5.2 (3.0)	5.0 (3.3)	5.9 (2.7)	5.7 (2.9)
Read books and magazines	71	4.9 (3.0)	4.5 (3.3)	5.3 (2.7)	4.8 (2.8)
Brew and drink coffee	50	3.5 (3.2)	3.1 (2.6)	3.8 (2.2)	3.8 (2.5)
Hum a tune	41	2.9 (2.5)	2.3 (1.9)	3.8 (2.1)	3.4 (2.2)
Take a long bath	36	3.6 (2.0)	3.9 (2.5)	4.4 (1.9)	4.5 (2.4)
Throw away something you don’t need from the drawer	33	4.3 (1.9)	3.9 (2.4)	3.6 (2.0)	3.5 (2.1)
Go to a coffee shop after lunch	27	8.4 (1.8)	8.3 (2.2)	8.4 (1.8)	8.3 (2.1)
Put some bath powder in the bathtub	24	2.9 (2.0)	3.3 (2.1)	4.1 (2.0)	4.4 (2.0)
Close your eyes for 3 min	21	2.9 (1.7)	2.6 (2.2)	2.7 (1.1)	2.5 (2.3)
Take a different route on the way back home	20	3.0 (1.4)	3.4 (2.3)	3.1 (1.1)	3.3 (2.2)

^a^SD: standard deviation.

**Table 4:** Behavioral activations: top 10 activities in mastery achieved and their frequencies.

Activity	Frequency (number of reports)	Mastery achieved, mean (SD^a^)
Test-drive a new car	3	9.7 (0.6)
Go to a coffee shop after lunch	27	8.3 (2.2)
Exercise	3	8.3 (2.9)
Call up an old friend	4	7.8 (2.9)
Go to yoga with a friend	6	7.2 (1.2)
Go to a hairdresser	3	6.7 (1.5)
Go to a gym	7	6.6 (3)
Walking	8	6.5 (0.8)
Get a haircut	4	6.5 (3)
Go to a meal with a friend	13	6.3 (2.8)

^a^SD: standard deviation.

**Table 5:** Behavioral activations: top 10 activities in pleasure achieved and their frequencies.

Activity	Frequency (number of reports)	Pleasure achieved, mean (SD^a^)
Test-drive a new car	3	9.7 (0.6)
Call up an old friend	4	9.0 (1.4)
Exercise	3	8.7 (2.3)
Go to a coffee shop after lunch	27	8.3 (2.1)
Go to yoga with a friend	6	7.8 (1)
Call up a family and hear their voice	5	7.4 (1.5)
Go to a meal with a friend	13	7.2 (2.3)
Go out for a luxurious lunch	17	6.8 (2.9)
Take a walk	8	6.6 (2)
Nail art	4	6.3 (3.8)

^a^SD: standard deviation.

Some activities brought greater levels of mastery and pleasure than initially expected. Such pleasant surprises included “Exercise,” “Do a makeup,” “Buy a comic book at a convenience store,” “Call up an old friend,” or “Go out for a luxurious lunch” (Tables 6 and 7).

### Cognitive Restructurings

All the cognitive restructuring items showed statistically significant reductions in sad or depressed, anxious or worried, or angry feelings when the emotion levels were compared pre-post within each situation that the patient worked on (Table 8). Typically, the level of emotion went down from approximately 3.5 to 2.1, showing a reduction greater than 1 point, on a scale of 0 to 5.

When the four tools were compared against each other, again within each situation, friend’s call and % Calculator both outperformed fact glasses. The average change in emotion level was −1.6 (SD 1.3), −1.5 (SD 1.3), −1.4 (SD 1.3), and −1.3 (1.3), respectively, for friend’s call, what-now microphone, % calculator, and fact glasses.

### Contrasts Between Beneficiaries and Nonbeneficiaries of Kokoro-App

The median of the final change score on PHQ-9 was 4. We therefore split the cohort into beneficiaries from Kokoro-App (change greater than 4: n=31) and nonbeneficiaries (change of 4 or less: n=49, including six who showed deterioration from baseline).

Although the beneficiaries tended to complete more sessions, need fewer days to complete one session, and spent more time per session, the group differences were not statistically significant (Multimedia Appendix 1).

Neither did the numbers of mind maps completed, overall and by emotion differ between the two groups, although the beneficiaries tended to report a slightly higher level of happy emotion.

**Table 6:** Behavioral activations: top 10 activities in unexpected mastery and their frequencies.

Activity	Frequency (number of reports)	Mastery achieved-expected, mean (SD^a^)
Exercise	3	1.3 (2.3)
Buy a comic book at a convenience store	4	1.0 (1.4)
Call up an old friend	3	1.0 (1.7)
Do a makeup	5	0.8 (1.3)
Call up a family and hear their voice	5	0.8 (1.3)
Go see a movie	4	0.8 (1.0)
Test-drive a new car	3	0.7 (0.6)
Nail art	3	0.7 (2.1)
Take a long bath	32	0.6 (1.7)
Put some bath powder in the bathtub	21	0.5 (1.0)

^a^SD: standard deviation.

**Table 7:** Behavioral activations: top 10 activities in unexpected pleasure and their frequencies.

Activity	Frequency (number of reports)	Pleasure achieved-expected, mean (SD^a^)
Do a makeup	5	1.0 (1.7)
Call up an old friend	3	1.0 (1.7)
Test-drive a new car	3	0.7 (0.6)
Go out for a luxurious lunch	15	0.6 (1.9)
Say hurray!	9	0.6 (0.7)
Borrow and watch a DVD	8	0.6 (2.7)
Go to a gym	7	0.6 (2.9)
Put some bath powder in the bathtub	21	0.5 (1.0)
Take a long bath	31	0.4 (1.2)
Throw away something you don’t need from the drawer	25	0.4 (1.7)

^a^SD: standard deviation.

**Table 8:** Changes in emotion levels by cognitive restructuring items. The statistical test was done with within-situation paired *t* test.

Item and emotion		Before, mean (SD^a^)	After, mean (SD)	Change, mean (SD)	*P* value
**Fact glasses**					
	Sad or depressed (n=68)	3.5 (1.3)	2.4 (1.3)	−1.5 (1.3)	<.001
	Anxious or worried (n=61)	3.7 (1.1)	2.2 (1.4)	−1.5 (1.2)	<.001
	Angry (n=44)	3.8 (1.2)	2.3 (1.3)	−1.1 (1.3)	<.001
**% Calculator**					
	Sad or depressed (n=48)	3.5 (1.3)	2.2 (1.2)	−1.3 (1.3)	<.001
	Anxious or worried (n=49)	3.7 (1.1)	2.1 (1.4)	−1.6 (1.2)	<.001
	Angry (n=33)	3.8 (1.3)	2.2 (1.1)	−1.5 (1.3)	<.001
**Friend’s call**					
	Sad or depressed (n=50)	3.6 (1.3)	2.1 (1.2)	−1.5 (1.3)	<.001
	Anxious or worried (n=46)	3.7 (1.2)	2.1 (1.2)	−1.7 (1.3)	*<*.001
	Angry (n=30)	3.4 (1.3)	2.0 (1.1)	−1.4 (1.3)	*<*.001
**What-now microphone**					
	Sad or depressed (n=41)	3.4 (1.3)	2.0 (1.2)	−1.4 (1.3)	<.001
	Anxious or worried (n=45)	3.9 (1.1)	2.1 (1.2)	−1.8 (1.2)	<.001	
	Angry (n=30)	3.6 (1.3)	2.4 (1.2)	−1.2 (1.3)	<.001

^a^SD: standard deviation.

The use of behavioral activation differed significantly in many aspects between the successful users and the less successful ones. The former conducted almost twice as many behavioral activations and expected and achieved greater levels of mastery or pleasure. The kinds of behavioral activation tasks chosen were significantly different: differences by more than 3% were found for activities such as “Listen to favorite music,” “Read books and magazines,” “Go to a coffee shop after lunch” (all more frequent among the beneficiaries), and “Take a long bath” (more frequent among nonbeneficiaries). The time categories of activities chosen were also different: the beneficiaries chose activities likely to require 60 min, whereas the nonbeneficiaries chose activities requiring 5 min or less.

The successful users of Kokoro-App also conducted more cognitive restructuring than the less successful ones, especially those using fact glasses and % Calculator. The decrease in emotion levels, however, was significantly different between the two groups only when using % Calculator.

## Discussion

### Summary of Findings

Kokoro-App was well accepted among the patients who had been unresponsive to one or more antidepressants and were moderately to severely depressed at baseline. The patients proceeded with the sessions in Kokoro-App at their own pace, spending approximately 60 min across 10 days for each session. Over the course of 9 weeks, on average, they completed 10 mind maps for self-monitoring own emotions and thoughts, conducted 14 behavioral activation personal experiments, and filled in six cognitive restructuring worksheets.

To the best of our knowledge, this study is the first study to examine specific details of behavioral activation or cognitive restructuring tasks conducted by the patients undergoing remote CBT or face-to-face CBT among a sizable number of clinical patients. Very interesting pictures emerged. Although patients often conducted activities with expected and achieved mastery or pleasure levels, around 4 to 5, a number of candidate activities emerged that achieved higher than expected mastery or pleasure. Such activities included, among others, “Test-drive a new car,” “Go to a coffee shop after lunch,” “Exercise,” “Call up an old friend,” “Do a makeup,” and “Go out for a luxurious lunch.” All the cognitive restructuring items were able to reduce the emotion levels significantly.

The study is also the first to compare details of the behavioral and cognitive skills practiced by the patients with regard to the outcome. The successful users of Kokoro-App conducted twice as many behavioral experiments of different kinds and of different time requirements than those who were less successful. The former also conducted significantly more cognitive restructuring tasks, especially using % Calculator.

### Limitations of the Study

These are several caveats in the interpretation of the current findings. First, although the original study was an RCT examining the value of adjunctive use of Kokoro-App, this study is by nature an observational study of the users of Kokoro-App. The current results therefore indicate association but not necessarily causation. The amount and nature of behavioral activation tasks or cognitive restructuring worksheets are potential mediators in the causative process from using the smartphone CBT to reduction in depressive symptomatology. It is possible that the beneficiaries of Kokoro-App got better because they engaged in more behavioral activations, or it is also possible that they conducted more behavioral activations because they had already felt better and had more energy. Second, in accordance with the observational nature of the study, we did not correct for multiple statistical testing and the analyses remain hypothesis-generating rather than confirmatory. The insights gained need be examined in future confirmatory experiments to provide ultimate guidance on how to conduct CBT. Third, strictly speaking, the findings only apply to Kokoro-App and the Japanese patients with moderate to severe depression on an antidepressant treatment. Whether they would apply to other smartphone or Internet CBT (iCBT), or whether they apply to Japanese nonclinical populations or to non-Japanese patients when they use Kokoro-App cannot be taken for granted. For example, “Take a long bath” or “Put some bath powder in the bathtub” may be particularly comforting for the Japanese people who traditionally take great pleasure in taking baths and may not necessarily apply to people living in different cultural traditions. The candidate activities list must certainly be culturally adapted when Kokoro-App is transferred to different countries. It must also be emphasized that app contents need be contextualized for each user’s age, sex, personal relationships, disabilities, and so on to make them more specific.

### Implications of the Study Findings

Nonetheless, our findings have important implications at three levels. First, they suggest how Kokoro-App can be improved in the next upgrade. Currently Kokoro-App lists the candidate activities according to the number of “Nice!”s that the patients have voted. This is probably a good feature of the app, creating an atmosphere of a therapeutic community. The next version of Kokoro-App can probably add another dimension to the recommendation by highlighting such activities that may not have been experimented by many but which turned out to produce great mastery or pleasure. The next version of Kokoro-App may also choose to emphasize % Calculator, and possibly do away with fact glasses as the latter has been found to be less effective than the other items. % Calculator and fact glasses aim to derive the same kinds of information, namely evidence for and evidence against the automatic thoughts but through different Socratic questions. % Calculator may be easier to understand for the users.

Second, they provide some insight on how iCBT and CBT in general can be better practiced. Our results suggest that behavioral activation best distinguishes the more from the less successful users of smartphone CBT. This finding is in line with a growing number of RCTs showing similar effectiveness of behavioral activation in comparison with the full CBT package, including cognitive restructuring [7-9]. However, these studies compared the different versions of CBT in the face-to-face settings. Whether the iCBT may as well consist only of behavioral activation or need to include cognitive restructuring is an empirical question warranting a direct randomized comparison.

Third, they also provide suggestions for the next generation of mobile health (mHealth). Providing the mHealth intervention on the Web or via a smartphone increases accessibility of the intervention but is only taking advantage of one aspect of the technology. The program can be used to collect valuable information of what the users of the program do or feel. It may also be combined with habit formation activities. Development of such an e-monitoring system has its own difficulties and complexities, including privacy, integration, and customization [21] but our Kokoro-Web presents one successful example and our study an example of how such a system enables collection of important and fertile information.

## Appendix

Multimedia Appendix 1 Beneficiaries and nonbeneficiaries of Kokoro-App.

Multimedia Appendix 2 FLATT investigators and committee members.

## Abbreviations

CBT: cognitive behavioral therapy
iCBT: Internet cognitive behavioral therapy
mHealth: mobile health
SD: standard deviation
