# Supplementary material for: Cognitive and Behavioral Skills Exercises Completed by Patients with Major Depression During Smartphone Cognitive Behavioral Therapy: Secondary Analysis of a Randomized Controlled Trial
Source: JMIR Ment Health. 2018 Jan 11;5(1):e4. doi: 10.2196/mental.9092 (PMC5785683; doi:10.2196/mental.9092)
Supplement: Multimedia Appendix 1 [file mental_v5i1e4_app1.pdf]

Multimedia Appendix 2

Beneficiaries and nonbeneficiaries of Kokoro-App

Characteristics			Beneficiaries (n=31), mean (SD[a])/ n (%)	Nonbeneficiaries (n=49), mean (SD)/ n (%)	*P* value
Sessions completed, mean (SD)			7.2 (6.8-7.6)	6.8 (6.3-7.2)	.17
Days taken to complete one session, mean (SD)			10.4 (8.8-11.9)	11.1 (9.8-12.3)	.50
Actual time (min) per session, mean (SD)			84.5 (35.2-133)	49.1 (31.7-66.3)	.11
**No. of mind maps, completed, mean (SD)**			9.5 (6.3-12.7)	12.2 (8.8-15.6)	.26
	No. of sad or depressed mind maps, mean (SD)		3.5 (2.4-4.7)	3.1 (1.9-4.2)	.57
	No. of anxious or worried mind maps, mean (SD)		3.6 (2.1-5.1)	2.7 (1.8-3.5)	.23
	No. of angry mind maps, mean (SD)		2.9 (1.4-4.4)	2.1 (1.3-2.9)	.31
	No. of happy mind maps, mean (SD)		2.2 (1.3-3.1)	1.8 (0.8-2.8)	.57
	Level of sad or depressed emotion, mean (SD)		3.6 (3.3-4.0)	3.5 (3.2-3.9)	.70
	Level of anxious or worried emotion, mean (SD)		3.6 (3.3-4.0)	3.5 (3.2-3.8)	.64
	Level of angry emotion, mean (SD)		3.6 (3.2-4.1)	3.4 (3.0-3.8)	.46
	Level of happy emotion, mean (SD)		3.6 (3.2-4.0)	3.0 (2.6-3.4)	.04
**No. of behavioral activations, completed, mean (SD)**			19.6 (11.3-28.0)	10.3 (6.9-13.7)	.02
	**Mastery or pleasure by behavioral activation**				
		Mastery expected, mean (SD)	5.1 (4.4-5.8)	3.9 (3.3-4.5)	.01
		Mastery achieved, mean (SD)	5.0 (4.3-5.7)	3.8 (3.3-4.4)	.01
		Pleasure expected, mean (SD)	5.4 (4.8-6.0)	4.2 (3.7-4.8)	.01
		Pleasure achieved, mean (SD)	5.3 (4.6-5.9)	4.3 (3.7-4.8)	.02
	**Top 10 activities chosen**				
		Listen to favorite music, n (%)	67 (9.8)	33 (5.8)	<.001

		Read books and magazines, n (%)	60 (8.8)	23 (4.0)	
		Brew and drink coffee, n (%)	25 (3.7)	27 (4.8)	
		Hum a tune, n (%)	24 (3.5)	19 (3.3)	
		Take a long bath, n (%)	12 (1.8)	29 (5.1)	
		Throw away something you don't need from the drawer, n (%)	16 (2.4)	20 (3.5)	
		Go to a coffee shop after lunch, n (%)	27 (4.0)	1 (0.2)	
		Put some bath powder in the bathtub, n (%)	17 (2.5)	12 (2.1)	
		Close your eyes for 3 min, n (%)	8 (1.2)	15 (2.6)	
		Take a different route on the way back home, n (%)	7 (1.0)	15 (2.6)	
	**Time categories of activities chosen**				
		Less than 5 seconds, n (%)	138 (20.2)	136 (23.9)	.002
		Less than 5 min, n (%)	211 (30.9)	210 (36.9)	
		Less than 60 min, n (%)	228 (33.4)	135 (23.7)	
		60 min or more, n (%)	105 (15.4)	88 (15.5)	
	Activities initiated by the patient, n (%)		234 (34.3)	157 (27.6)	.01
	Activities recommended by the patient, n (%)		40 (5.9)	16 (2.8)	.01
**Cognitive restructuring, no. completed per person, mean (SD)**			8.0 (5.2-10.9)	5.2 (3.8-6.6)	.05
	**Cognitive restructuring items used, per person**				
		No. using fact glasses, mean (SD)	2.8 (1.9-3.7)	1.6 (1.1-2.0)	.01
		No. using % Calculator, mean (SD)	2.2 (1.3-3.1)	1.1 (0.8-1.4)	.01
		No. using friend's call, mean (SD)	1.7 (1.1-2.3)	1.3 (0.9-1.8)	.30
		No. using what-now microphone, mean (SD)	1.6 (1.0-2.3)	1.2 (0.8-1.7)	.27
		Change in emotion level by fact glasses, mean (SD)	1.4 (1.1-1.7)	1.3 (1.0-1.5)	.45
		Change in emotion level by % Calculator, mean (SD)	1.8 (1.4-2.2)	1.3 (0.9-1.6)	.04

		Change in emotion level by friend’s call, mean (SD)	1.9 (1.5-2.4)	1.4 (1.0-1.8)	.10
		Change in emotion level by what-now, mean (SD)	1.8 (1.3-2.3)	1.5 (1.1-1.9)	.36

[a]SD: standard deviation.
